# Supplementary material for: Predictors of perinatal death in the presence of missing data: A birth registry-based study in northern Tanzania
Source: PLoS One. 2020 Apr 16;15(4):e0231636. doi: 10.1371/journal.pone.0231636 (PMC7161952; doi:10.1371/journal.pone.0231636)
Supplement: Attachment — Submitted filename: review comments_plosone.docx [file pone.0231636.s001.docx]

**Reviewed comments from Unnati Saha**

The manuscript “Predictors of perinatal death in the presence of missing data: A registry-based study in northern Tanzania”

The paper focused on an important topic to address on the determinants of perinatal mortality and importance of missing observations and their attribution in the analysis. My comments are given below.

-Overall the paper is written well, but additional revision by an expert English editor is necessary for the improvement of the manuscript for publication in Plos One.

Specific comments are given as below.

**Abstract:**

**Backgorund**

-perhaps it would be complete if author(s) replace “zonal”with the complete name of the hospital e.g., Kilimanjaro Christian Medical centre (KCMC).

**Methods**

-In this section Author(s) probably can mention KCMC instead writing the full name of the hospital

**Conclusion**

- “Appropriate choice of the analysis model should be considered in similar studies”. Not clear about this sentence.
- Which one is benchmark model? Please refer the benchmark model based on results
- Also, better if author(s) revise the last two sentences under the conclusion section.

**Main Body text**

**Overall comments**

**-** Manuscript does not include page numbers and line numbers, which hinders to reporting reviewed comments orderly

**Introduction:**

- paragraph third, last sentence, I understand that the author(s) performed analysis with and without missing values imputation and compared the results for statistical significance. So, perhaps it would be better if they explain the aim of the study completely.

**Methods:**

**Study design and participants**

-second paragraph, Author(s) did not make it explicit why they excluded multiple births from the analysis

-the explanation of the method how missing values were imputed are missing, also better give a theoretical explanation of the use of two methods how one model better perform over other model.

**variables**

- author(s) included so many variables in the analysis, so I am wondering whether it hampers the effects of some variables on estimated perinatal mortality due to co-linearity. For example, low birthweight, gestational week, preterm birth, and other medical indicators. So, author(s) need to be cautious about entering variables in the model.

-also, as WHO recommended for 8 ANC visits (I read in the INTRODUCTION section), author(s) perhaps could try to investigate the effects of 8 visits as well.

- I miss the definition of the perinatal death (as dependent variable) whether dummy or continuous, although next section statistical analysis it gives a hint.

**Statistical analysis**

- first paragraph, the explanation of the method how missing values were imputed are missing.

- Model I (‘binomial family’ means outcome variable is coded as dummy), Model II (log-linear regression with passion family) means outcome variable is count variable. How they handle the 0 counts (no perinatal deaths) in the analysis.

- Author(s) performed two models using two types of outcomes. Author(s) could play with variables in different models of both outcome variables “perinatal mortality”, e.g., child, mother, family, clinical etc. different levels of variables. The results could provide better understanding of the effects on the model outcome.

- author(s) applied GEE method, but as author(s) expect multiple deaths born to a mother, so how the analysis handled the correlated deaths in one family (mother)?

- if deaths are correlated and not accounted for this, it may spurious the conclusion on determinants of perinatal deaths. Author, may give explanation on this.

- again, perhaps author(s) can explain imputation technique in better way for general readers.

**Results:**

**Maternal background and characteristics at first birth**

- Why first birth? I understand that author(s) analyzed perinatal mortality that occurred at any birth born to a mother in the hospital and also in rural area referred to this hospital
- Few cases (<=.5%) are recorded against variables PPH, Abruption placenta, placenta previa, so I am wondering whether entering those variables in the model will be appropriate. It may miss-specify the model. Also, better if there is an explanation how those variables can effect pregnancy and mortality e.g., PROM.
- Also check all variables distribution including alcohol drinking for the referral cases as analysis was stratified by referral vs not referral cases.
- Table 3, should it be “maternal characteristics”? revise the title

**Predictors of perinatal death**

-Table 4, n should be different before and after imputation, so better to mention them

- do the author(s) have any explanation how alcohol consumption may reduce perinatal deaths (see Table 4)? Also, explain the results of alcohol consumption in model 5 for referral cases vs. not referral cases.

-Table 5, which model employed for this analysis? Seeing the sample size it seems model with imputation data, but not clear which model (from the title)? Model I or Model II?

- Seeing Table 5 and coefficients of alcohol use it seems to me that better exclude this variable from the analysis. The effects are different for referral and not referral, and that is the reason that in Model II in Table 4, after imputation the effect was lower and insignificant.
- Table 6, again correct the number of cases
- Women had complication and that is why probably they had CS delivery. It’s effect may affect the effects of referral including other complication variables.
- Better to add a section explaining model and better fitted model

- Discussion section requires careful revision with proper explanation of the effects with references and conclusion drawn from this study.

I would suggest to accept the paper after incorporating the suggested comments indicated as above of this note.
