# Supplementary material for: Predictors of perinatal death in the presence of missing data: A birth registry-based study in northern Tanzania
Source: PLoS One. 2020 Apr 16;15(4):e0231636. doi: 10.1371/journal.pone.0231636 (PMC7161952; doi:10.1371/journal.pone.0231636)
Supplement: Attachment — Submitted filename: Response to Reviewers.docx [file pone.0231636.s002.docx]

P. O. Box 2240,

Moshi – Tanzania.

23 March 2020.

The Journal Editor,

Plos One.

Dear Editor,

**Re: Response to reviewer’s reports for the manuscript with ID number PONE-D-19-33574**

Thank you very much for the invitation to resubmit our manuscript entitled: **“Predictors of perinatal death in the presence of missing data: A registry-based study in northern Tanzania.”** We appreciate the editorial and reviewer’s useful comments and suggestions to help improving our manuscript. We have addressed all the comments and suggestions raised by the reviewers and editors and we hope that our manuscript will now be acceptable for publication.

Please find below our responses indicated in red-colored fonts. All changes in the main text are in track changes. When referring to page and line numbers for reference purposes, we are referring to the document with track changes.

Regards,

Innocent B. Mboya

Corresponding author

**Journal requirements:**

1. When submitting your revision, we need you to address these additional requirements.

Please ensure that your manuscript meets PLOS ONE's style requirements, including those for file naming. The PLOS ONE style templates can be found at

<http://www.journals.plos.org/plosone/s/file?id=wjVg/PLOSOne_formatting_sample_main_body.pdf> and <http://www.journals.plos.org/plosone/s/file?id=ba62/PLOSOne_formatting_sample_title_authors_affiliations.pdf>

We have revised the entire manuscript to make sure it meets the journal requirements.

2. Please amend your current ethics statement to address the following concerns: Please explain why was written consent was not obtained, how you recorded/documented participant consent, and if the ethics committees/IRBs approved this consent procedure.

We thank the editor for this important observation. The ethics statement has been updated. Since the interview was administered just after the woman had given birth, consent was given orally. These changes are reflected in the ethical consideration section, page 11, lines 244-246. The ethical procedure, including the oral consent, was approved by the local Institutional Review Board of KCMC hospital and the National Ethics Committee in Norway.

3. We note that you have indicated that data from this study are available upon request. PLOS only allows data to be available upon request if there are legal or ethical restrictions on sharing data publicly. For information on unacceptable data access restrictions, please see <http://journals.plos.org/plosone/s/data-availability#loc-unacceptable-data-access-restrictions>.

In your revised cover letter, please address the following prompts:

a) If there are ethical or legal restrictions on sharing a de-identified data set, please explain them in detail (e.g., data contain potentially identifying or sensitive patient information) and who has imposed them (e.g., an ethics committee). Please also provide contact information for a data access committee, ethics committee, or other institutional body to which data requests may be sent.
b) If there are no restrictions, please upload the minimal anonymized data set necessary to replicate your study findings as either Supporting Information files or to a stable, public repository and provide us with the relevant URLs, DOIs, or accession numbers. Please see <http://www.bmj.com/content/340/bmj.c181.long> for guidelines on how to de-identify and prepare clinical data for publication. For a list of acceptable repositories, please see <http://journals.plos.org/plosone/s/data-availability#loc-recommended-repositories>.

We will update your Data Availability statement on your behalf to reflect the information you provide.

The KCMC medical birth registry data contains potentially identifying and sensitive patient information. This has also been stipulated by the local Institutional Review Board of KCMC hospital and the National Ethics Committee in Norway when establishing this birth registry. Permission to use the data in this study was made through the Kilimanjaro Christian Medical University College Research and Ethics Review Committee, and received an approval number 2424. The authors do not have the legal right to share the data publicly. All data requests can be sent directly to the Executive Director of the KCMC referral hospital, P. O. Box 3010, Moshi, Tanzania or through the corresponding author.

4. Thank you for stating the following in the Acknowledgments Section of your manuscript:

"We would like to acknowledge the midwives who participated in data collection as well as all women whose information enabled availability of data used in this study. We also acknowledge the Norwegian Council for Higher education program and Development Research (NUFU) for funding the establishment of the KCMC Medical birth registry. This funding was not related to this manuscript.".

We note that you have provided funding information that is not currently declared in your Funding Statement. However, funding information should not appear in the Acknowledgments section or other areas of your manuscript. We will only publish funding information present in the Funding Statement section of the online submission form.

* Please remove any funding-related text from the manuscript and let us know how you would like to update your Funding Statement. Currently, your Funding Statement reads as follows:

"This work was supported through the DELTAS Africa Initiative Grant No. 107754/Z/15/Z-DELTAS Africa SSACAB. The DELTAS Africa Initiative is an independent funding scheme of the African Academy of Sciences (AAS)’s Alliance for Accelerating Excellence in Science in Africa (AESA) and supported by the New Partnership for Africa’s Development Planning and Coordinating Agency (NEPAD Agency) with funding from the Wellcome Trust (Grant No. 107754/Z/15/Z) and the UK government. The views expressed in this publication are those of the author(s) and not necessarily those of AAS, NEPAD Agency, Wellcome Trust or the UK government.

The Norwegian Council for Higher Education’s Program for Development Research (NUFU) funded the establishment of the birth registry at the Kilimanjaro Christian Medical Center (KCMC).

The funders had no role in study design, data collection, and analysis, decision to publish, or preparation of the manuscript."

We thank the editor for this comment. We are very sorry for this confusion; we have now revised the acknowledgment section and also removed the funding information. Changes are on page 32, lines 604-609.

Please update the funding information to include all the text in green color above. Additional information is in the second paragraph of the green text above.

**Reviewer reports:**

**Reviewer #1:**

Thank you for the invitation to review this manuscript, which I read with great interest. The authors used data from KCMC medical birth register, which collected information from mothers about their health during pregnancy, delivery details and characteristics of the child. The authors used these data to explore risk factors for perinatal deaths (stillbirths and early neonatal deaths), applying multiple imputation to account for missing data. The paper looks at an interesting subject, but some key information about missing data is not reported (especially given it’s the key focus of the paper), and I have some concerns about selection of variables in the model. Major revisions are required prior to publication.

**Major comments:**

Given that evaluation of the impact of missing data is one of the key aims of the paper, there is insufficient information about missing data, patterns and imputation. The authors should consider adding information on:

1. The patterns of missing data: how much data was missing and for which variables? the authors state in tables 1-2 that counts don’t add up to the total, but the number of pregnancies with missing data should be added. It is not clear how many children were included in the cohort overall and how many in the complete case cohort for analyses?

We thank the reviewer for his suggestion/ comment. We have provided more details about the analysis of missing data in the Statistical analysis section, page 10, line 207 to page 11, line 241. This includes information about the variables that were imputed. Results about the number and proportions of missing values have also been provided in Table 3, page 15. We chose to present these results in a separate table for ease of visualizing all the variables with missing values showing the percentage distribution of their contributions.

Regarding the number of children included in the analysis, we would like to indicate that the unit of analysis was a child (i,e, singleton deliveries). Fig 1 has been revised to provide a clearer description of the overall number of children included in the analysis. As indicated in this figure, we analyzed data for a total of 50,847 deliveries, which also contained variables with missing values, including perinatal status. The data was multilevel type where we modelled singleton deliveries nested within a mother.

2. What was the impact of missing data on the perinatal mortality? It is not clear what was the perinatal mortality rate in the full cohort (including all pregnancies with missing data) compared to complete case cohort. It is unclear why the mortality rate went up after imputation (from 41.6/1000 births to 53/1000 births)

We thank the reviewer for this very important comment. After re-examining the analysis, we noted a mistake in the analysis in which the tabulation of perinatal status variable did not account for missing values. By this we mean that, instead of using *mi estimate: prop varname,* we initially used *tab varname* commands in Stata. The latter is incorrect as it does not take in to account the imputed data. We have corrected this text from the results paragraph of the abstract, page 2, line 42-44, and the results section in page 18, line 314-315. The discussion has also been updated to reflect these changes. By making these changes, the PMR remained the same even after imputation of missing values.

Yet, we would like the reviewer to note that, in any situation where the missing data is imputed, the parameter in question, whether prevalence, incidence, mean etc. is likely to change, often increase because the analysis is now capable of using full information about the variable. The aim of imputation is to reduce the bias inherent by using complete case analysis.

3. What was the imputation model? Which variables needed to be imputed and which were used to predict missing data?

We appreciate the reviewer for raising these essential questions. We have addressed these questions by providing more details about the imputation models as well as data analysis, as indicated above, including which variables needed to be imputed.

4. Authors state in multiple parts of the manuscript that complete case analyses were biased and led to incorrect conclusions. However, the results seem to be largely consistent between complete case and imputed data, with slightly different effect sizes, but the same conclusions would be reached based on complete case data. I think such strong statements should be avoided

We thank the reviewer for this relevant comment. We have now revised the discussion and conclusions to address a concern about strong statements used to compare our findings and make conclusions. As the reviewer has also indicated, the direction of association between variables presented in the now Table 5 on the risk of perinatal death is largely the same for both the complete case and the imputed datasets. However, we would like to indicate that the coefficients resulting from complete case analysis are observed to be either slightly higher or lower than should have been if there were no missing values. Also, standard errors for the complete case analysis are all higher than that of the imputed data. The shared paper below also points out clearly that, under the missing at random assumption, multiple imputation corrects such biases that may arise in complete case analyses.

The authors might find these suggested reporting guidelines for papers using multiple imputation useful: Sterne JA, White IR, Carlin JB, et al. Multiple imputation for missing data in epidemiological and clinical research: potential and pitfalls. BMJ. 2009; 338:b2393. Published 2009 Jun 29. doi:10.1136/bmj.b2393

This is a very important paper, and we thank the reviewer for sharing. It has provided an important insight for describing the model-building strategies of the imputation models. We have addressed the key issues discussed in the paper. For instance, we revised the data analysis section while also comparing results before and after imputation of missing values, even in the initial draft.

My second major comment related to the modelling strategy. What was variable selection strategy? It looks that all risk factors available were included in the model, but they are likely to be collinear, eg having various pregnancy complications is probably closely correlated with referral for delivery. Having multicollinearity in the model could introduce bias to the analysis, e.g. the odd result for alcohol consumption during pregnancy (showing protective effect) could be a result of that over adjustment in presence of other unmeasured confounding. The authors should have a look at literature on birthweight paradox.

Stepwise regression was used for model building and variable selection. Before the regression analysis, we used pair wise correlation matrix to assess the linear relationship between variables to be entered in the regression models. We did not find a strong linear relationship/ correlation between these variables. Even after fitting the regression models, the collinearity statistics i.e. VIF and tolerance (1/VIF) agreed with these results. Therefore, multicollinearity was not a problem in our analysis.

We thank the reviewer for the comment about looking at literature on the birth weight paradox. Indeed, this might be the case in this study for the association between alcohol consumption and adverse pregnancy outcomes. As we have also described in the discussion section, page 29, line 518-522, it is unlikely that alcohol consumption itself can explain the unobserved association which also agrees with findings from a study using the same dataset (between the year 2000 and 2010) by Isaksen et al., 2015.

**Other comments:**

1. Perinatal mortality should be defined as stillbirths and early neonatal deaths per 1000 total births, including live and stillbirths (see WHO ICD10 instruction manual https://www.who.int/classifications/icd/ICD10Volume2_en_2010.pdf?ua=1), but the authors report perinatal mortality per 1000 live births

We thank the reviewer for this observation. We used the same definition but we have corrected this issue in the manuscript by deleting the word “live” when defining perinatal death.

2. Abstract: explain all abbreviations as it is not obvious what they are

All the abbreviations have been explained in the abstract.

3. Methods: Why did the authors run models to estimate both odds ratios and risk ratios, rather than just risk ratios?

The purpose of running separate models to estimate both odds and risk ratios was only for comparison purposes. Our interest was to show the performance of these models in estimating the effect of covariates on perinatal outcomes. What we have observed is that the GEE model estimating odds ratios slightly overestimates the effects as opposed to the GEE models that estimated risk ratios. However, we are open to the reviewer’s comment on how best to report such findings.

4. Methods: The authors note that mothers could chose not to participate - what proportion of mothers opted out and hence is not covered?

This is an important issue raised by the reviewer. Unfortunately, we cannot get this information from the dataset.

5. Results: are figures 1 and 2 showing percentage out of all births? Since the authors have fitted a trend, by how much did perinatal mortality decline? The results splitting perinatal mortality into stillbirths and early neonatal deaths could be discussed more – the declines were driven by early perinatal mortality as stillbirth rate remained constant, but in most recent years something has changed and early neonatal deaths went up while stillbirth went down. Why is that? Is it due to registration practices? It would be worth discussing.

Figure 1 and 2 shows percentage out of all births in each respective year, and not the overall number of births. Results on how much the proportion of perinatal death has declined between 2000 and 2015, are in the results, trends of perinatal death section, page 18, line 314-315. Furthermore, we have also described the results on the changes in the proportion of early neonatal deaths and stillbirth in recent years i.e. from 2013 to 2015. These changes are reflected in the results, trends of perinatal death section, page 18, line 316-320, and discussion section, page 19, line 25-29. A potential reason for the observed trends is registration practices as indicated by the reviewer, and has also been reported elsewhere by Bergsjo et. al., 2010 on the “Recording of maternal deaths in an East African university hospital”. The authors used the same dataset but between the years 2000-2007.

**Reviewer #2**:

The manuscript “Predictors of perinatal death in the presence of missing data: A registry-based study in northern Tanzania”. The paper focused on an important topic to address on the determinants of perinatal mortality and importance of missing observations and their attribution in the analysis. My comments are given below.

- Overall the paper is written well, but additional revision by an expert English editor is necessary for the improvement of the manuscript for publication in Plos One.

We acknowledge the reviewer's comment. The manuscript has been revised as recommended.

Specific comments are given as below.

**Abstract:**

Background

- perhaps it would be complete if author(s) replace “zonal”with the complete name of the hospital e.g., Kilimanjaro Christian Medical centre (KCMC).

We have made the suggested changes in the Abstract section, line 29-30.

Methods

- In this section Author(s) probably can mention KCMC instead writing the full name of the hospital

We have deleted the full name of the hospital and used respective abbreviations. Changes are in the Abstract section, line 32.

Conclusion

- “Appropriate choice of the analysis model should be considered in similar studies”. Not clear about this sentence.

We acknowledge the reviewer's comment. Although we have revised the sentence, we have considered deleting this sentence to shorten the abstract.

- Which one is benchmark model? Please refer the benchmark model based on results

The benchmark model is the GEE log-linear model (Poisson Family, log-link function) with an exchangeable correlation structure and robust variance estimator. Although the direction of the associations is the same, the other GEE model slightly over- or under-estimated the coefficients and other parameters. The final results and conclusion are based on this model.

- Also, better if author(s) revise the last two sentences under the conclusion section.

The two sentences have been revised for clarity.

**Main Body text**

**Overall comments**

- Manuscript does not include page numbers and line numbers, which hinders to reporting reviewed comments orderly

We acknowledge the reviewer comment. The page and line numbers have been provided in the document with track changes. In all our revisions, we have made sure we included a specific page and line numbers where such changes are done.

Introduction:

- paragraph third, last sentence, I understand that the author(s) performed analysis with and without missing values imputation and compared the results for statistical significance. So, perhaps it would be better if they explain the aim of the study completely.

We acknowledge the reviewer's comment. The suggested changes are in the introduction section, page 5, lines 107-108.

Methods:

Study design and participants

- second paragraph, Author(s) did not make it explicit why they excluded multiple births from the analysis

We have provided additional information about why we excluded multiple births. Changes are in study design and participants section page 6, lines 134-135.

- The explanation of the method how missing values were imputed are missing, also better give a theoretical explanation of the use of two methods how one model better perform over other model.

This is an important observation by the reviewer. We have provided more details about missing values, missing data mechanisms, missing data patters and the imputation procedures. These changes are reflected in the Statistical analysis section, page 10, line 207, to page 11, line 241.

variables

- author(s) included so many variables in the analysis, so I am wondering whether it hampers the effects of some variables on estimated perinatal mortality due to co-linearity. For example, low birthweight, gestational week, preterm birth, and other medical indicators. So, author(s) need to be cautious about entering variables in the model.

We acknowledge the reviewer for this relevant comment. The first reviewer also raised the same issue. Indeed, a regression model with many variables is likely to suffer from multicollinearity. However, we would like to indicate here that we have tested for the presence of multicollinearity using both pair wise correlation (before fitting the models) and the collinearity statistics i.e. VIF and tolerance (1/VIF) after fitting the models. Based on these results, we did not find any evidence of multicollinearity, and so it was not a problem in this study. In addition, stepwise regression was also used to aid in variable selection. Furthermore, most of the factors included in this analysis are consistently reported in the existing literature to be associated with the risk of perinatal death.

- also, as WHO recommended for 8 ANC visits (I read in the INTRODUCTION section), author(s) perhaps could try to investigate the effects of 8 visits as well.

We thank the reviewer for this comment. We would like to first indicate that, data analyzed in this study were between the years 2000 and 2015 while the new WHO recommendations were issued on 2016. During this time (2000-2015), it is the at least 4 ANC visits guideline which was still in place. Furthermore, we tried to assess the effect of 8 ANC visits (only on the complete records) and observed almost no differences with 4 ANC visits. These descriptions are in the predictors of perinatal death section, page 20 lines 359-369.

- I miss the definition of the perinatal death (as dependent variable) whether dummy or continuous, although next section statistical analysis it gives a hint.

We have revised and provided a clear definition of perinatal death in the study variables and variable definitions section, page 8, line 161-167.

Statistical analysis

- First paragraph, the explanation of the method how missing values were imputed are missing.

More information about the analysis of missing data is reflected in the statistical analysis section, page 10, line 207, to page 11, line 241.

- Model I (‘binomial family’ means outcome variable is coded as dummy), Model II (log-linear regression with passion family) means outcome variable is count variable. How they handle the 0 counts (no perinatal deaths) in the analysis.

We appreciate the reviewer's comment. In both cases, the outcome is binary (1 if an event has occurred and 0 if otherwise). For the GEE log-linear regression model, i.e., Poisson family and log link function, the variance estimated by this model will be very close to the binomial variance when the outcome is rare (as is the case in this study). This model estimates risk in cohort studies and includes an additional over-dispersion parameter that penalizes the standard errors (in a situation where there are rare events), for more precise estimates.

For instance, Table 5 in page 22, if we compare the GEE log-binomial/ logit (Model I) and the GEE log-linear (Model II) regression models of the complete case analysis we see that, the estimates and confidence intervals for abruption placenta are (OR=19.469, 95%CI 12.357, 30.673) and (RR=4.472, 95%CI 3.552, 5.629), respectively. The former has very wide confidence intervals as compared to the later. This also justifies the need to simultaneously run two separate models, to estimate OR and RR to show such differences.

Back to the reviewer question on how we handled the zero counts, we would like to indicate that while there are methods to handle such values, we did not go as far as applying such regression models in this analysis. We will, however, consider this issue in further work.

- Author(s) performed two models using two types of outcomes. Author(s) could play with variables in different models of both outcome variables “perinatal mortality”, e.g., child, mother, family, clinical etc. different levels of variables. The results could provide better understanding of the effects on the model outcome.

We thank the reviewer for this critical comment. As we stated above, the outcome analyzed in this study is the same i.e., a binary variable, i.e., perinatal status. When developing the models in this analysis, we used stepwise regression for variable selection. We further performed a series of analyses by entering and removing different combinations of variables from each model until we arrived to the final results in this manuscript. We have provided a description in the statistical analysis section, page 10, line 209-210.

- author(s) applied GEE method, but as author(s) expect multiple deaths born to a mother, so how the analysis handled the correlated deaths in one family (mother)?

We acknowledge the reviewer's comment about the clustering of events within mothers. The GEE models account for correlation by a specification of the working correlation structure. In our analysis, we took care of this, although limited to the exchangeable correlation structure due to the unbalanced nature of the birth registry data. By unbalanced, we mean that there are women with one, two, three up to six children, in which case the more complex correlation structures (such as unstructured and autoregressive correlations) would not be compatible with such imbalances. The model with unstructured correlation did not converge; hence we could not trust and report such results.

- if deaths are correlated and not accounted for this, it may spurious the conclusion on determinants of perinatal deaths. Author, may give explanation on this.

This is a critical point raised by the reviewer. However, as described above, the GEE models are designed to handle the correlation of observations within clusters through the specification of the working correlation structure. In that respect, the addresses the problem of correlated observations.

- again, perhaps author(s) can explain imputation technique in better way for general readers.

As also explained above, we have provided more details about the analysis of missing data in the statistical analysis section, page 10, line 207, to page 11, line 241. We hope that these revisions will address reviewer’s concerns.

Results:

Maternal background and characteristics at first birth

- Why first birth? I understand that author(s) analyzed perinatal mortality that occurred at any birth born to a mother in the hospital and also in rural area referred to this hospital

The purpose of describing maternal characteristics at first birth is to describe the *baseline* characteristics of mothers at first recorded pregnancy in the KCMC birth registry, who contributed to total deliveries analyzed in this study.

- Few cases (<=.5%) are recorded against variables PPH, Abruption placenta, placenta previa, so I am wondering whether entering those variables in the model will be appropriate. It may miss-specify the model. Also, better if there is an explanation how those variables can effect pregnancy and mortality e.g., PROM.

We do agree with the reviewer's comment about the inclusion of these variables and their effects on the models, given their low prevalence. Indeed, as we have shown for the case of the abruption placenta, the confidence intervals are overly wide. However, as opposed to the binomial regression model, the modified Poisson also referred as the log-linear model (Model II) accounts for the overdispersion of data due to the low prevalence of these conditions. Results in Table 5, page 22 for Model II (both before and after imputation of missing values) show much narrower confidence intervals than for Model I.

We have briefly described the effect of PROM on pregnancy and mortality in the discussion section page 31, line 565-566.

- Also check all variables distribution including alcohol drinking for the referral cases as analysis was stratified by referral vs not referral cases.

We have re-checked the distribution of variables by referral status. For alcohol use during pregnancy for example, 18% of 13,260 were referred for delivery compared to 25.3% of 35,093 those who were not referred. The Chi-square statistic shows a statistically significant difference (p<0.001).

- Table 3, should it be “maternal characteristics”? revise the title

Table 3 (now Table 4, page 17) shows the distribution of perinatal status by maternal characteristics during pregnancy and delivery. We have not been able to figure out clearly what the reviewer would like us to do in this case. Please provide us with more details for us to revise and improve.

Predictors of perinatal death

- Table 4, n should be different before and after imputation, so better to mention them

We have revised the ‘n’ in now Table 5 (page 22) to reflect the actual numbers before and after imputation of missing values.

- do the author(s) have any explanation how alcohol consumption may reduce perinatal deaths (see Table 4)? Also, explain the results of alcohol consumption in model 5 for referral cases vs. not referral cases.

We acknowledge the reviewer’s observation on the protective effect of alcohol consumption on the risk of perinatal death in this cohort. These results were also unexpected to us. A previous study from the same dataset but between the years 2000 and 2010 (Isaksen et al., 2015) found a similar association. We have revised the discussion section, page 29, line 518-522 to describe the unlikely association between alcohol use and perinatal death in this cohort.

- Table 5, which model employed for this analysis? Seeing the sample size it seems model with imputation data, but not clear which model (from the title)? Model I or Model II?

Below each table with results from the regression analysis, we have provided a description of the analysis model used. For clarity, we have also provided the description for this table when describing the results in the stratified analysis by referral status section, page 23 line 408-409.

- Seeing Table 5 and coefficients of alcohol use it seems to me that better exclude this variable from the analysis. The effects are different for referral and not referral, and that is the reason that in Model II in Table 4, after imputation the effect was lower and insignificant.

We thank the reviewer for this comment. Despite the protective effect of alcohol consumption during pregnancy on the risk of perinatal death in several analysis, we still believe it is worth reporting such information. As earlier indicated, previous study from the same dataset has reported a similar association. The observed association does not necessarily refer causation. Other factors could be in the causal pathway in this association. We have revised the discussion section to capture this issue.

- Table 6, again correct the number of cases

Number of cases have been revised.

- Women had complication and that is why probably they had CS delivery. It’s effect may affect the effects of referral including other complication variables.

We agree that women with complications could be the ones who had CS delivery. Throughout our analysis, CS delivery was protective of the risk of perinatal death. Regarding referrals, we should point out that, more than three-quarters of all women were not referred for delivery. This means that they were probably visiting the KCMC hospital for both routine antenatal care services and delivery. Given the variation in the composition of the population in this cohort, it is less likely referral status to have affected the observed associations. Our findings, particularly in Table 6, page 24 shows that, most of the covariate effects in the group of women not referred for delivery showed a stronger association with the risk of perinatal death.

- Better to add a section explaining model and better fitted model

We acknowledge the reviewer’s comment. However, the first paragraph of predictors of perinatal death section, page 19 was intended to describe the better fitted model. For clarity, we have slightly revised this section and included a better comparison of results from different models clearly indicating the one used to interpret our findings.

- Discussion section requires careful revision with proper explanation of the effects with references and conclusion drawn from this study.

We acknowledge the reviewer’s comment. We have made revisions to the discussion section as recommended.

I would suggest to accept the paper after incorporating the suggested comments indicated as above of this note.

We thank the reviewer for this recommendation. We hope that the manuscript can now be accepted considering the changes were have made.
